# Quiet please: reducing occupational exposure to harmful noise

**DOI:** 10.2471/BLT.19.021019

**Published:** 2019-10-01

## Abstract

Making workers aware of the risks they incur is key to implementing hearing preservation initiatives, but some occupations present intractable challenges. Gary Humphreys reports.

People’s disregard for the fragility of their ears is a source of constant surprise to Dr Shelly Chadha. “People put on sunglasses to protect their eyes from ultraviolet light, but you rarely see people wearing ear plugs,” she says. “In fact, the tendency is to wear ear phones to play amplified music, often at harmful levels.”

Chadha, who heads up the World Health Organization’s (WHO) work on deafness prevention, believes that one of the reasons for this discrepancy is a failure to appreciate just how fragile ears are.

“The threshold at which damage starts to occur to hearing is just 85 decibels,” she says. “That level can easily be reached in a noisy restaurant or in a street where there is heavy traffic or construction.”

Noise-induced hearing loss usually begins with declining sensitivity to sound at high frequencies and typically shows up on audiograms as a dip or ‘notch’ at 4000 Hertz. “If noise exposure continues, this notch gradually deepens and spreads as the hair cells in the cochlea and the cells that support them deteriorate,” Chadha explains. “Once the damage is done there is no going back.”

Notwithstanding the challenges epidemiologists face in characterising the disease burden imposed by excessive noise, it is widely assumed that most noise-induced hearing loss occurs in the workplace. “Noise is present to some degree in almost all workplaces,” says Dr Ivan Ivanov, team lead, Global Occupational and Workplace Health at WHO headquarters. “It can be caused by a sudden sound, such as the bang of a rivet gun or by continuous exposure to loud sounds over an extended period, such as the noise generated by an assembly line.”

The main approaches to preventing noise-induced hearing loss are: to reduce the noise generated in the workplace, for example by investing in quieter machinery or moving machines away from where employees are normally located (referred to as engineering controls), to organize work in a way that limits worker exposure to noise (referred to as administrative controls) and to protect people’s ears by ensuring they wear earplugs or other protective devices.

“The priority is to eliminate the noise hazard,” says explains Dr Manal Azzi, an occupational safety and health specialist at the International Labour Organization (ILO). “If that is not possible, efforts should focus on engineering and administrative controls to mitigate the harm for workers, such as limiting the time they are exposed to the noise hazard. Relying on personal protective equipment should be a last resort.”

In recent years, workplace hearing preservation initiatives have been supported by technology, including personal sound exposure meters. The size of a bottle cap, these dosemeters are usually worn on the shoulder and measure the noise levels workers are exposed to throughout their shift, allowing employers to establish which areas may present a noise hazard and/or which employees are most exposed.

“Once the damage is done there is no going back.”

Shelly Chadha.

Wall-mounted sound monitoring devices, featuring luminous panels with icons corresponding to different sound levels, are also used to raise awareness among workers regarding their exposure and thus encourage noise reduction or the use of hearing protection. For example, such devices are being used by the car manufacturer Audi at worker training facilities in Ingolstadt and Neckarsulm, Germany and have been used in a critical care unit in Preston, England.

Unfortunately, many occupations preclude the elimination of noise sources, and have a limited scope for shielding workers from noise. Construction workers are among those with the highest levels of noise-induced hearing loss.

In most high-income countries hearing conservation measures are backed by regulation, that derives from ILO’s Working Environment (Air Pollution, Noise and Vibration) Convention of 1977 (updated in 2001) and includes the United States of America’s (USA) Occupational Noise Exposure Standard and the European Union’s (EU) Physical Agents Noise Directive.

In low-income countries the regulatory environment is less supportive of hearing conservation efforts, while the informal sector is largely unregulated. “Most informal sector workers lack social protection, rights at work and decent working conditions,” Ivanov says, “and that includes working conditions safe for people’s hearing.”

One occupation often criticized in this regard is ship recycling. Previously located in Taiwan, China and the Republic of Korea, the bulk of ship recycling is now carried out in Bangladesh, China, India, Pakistan and Turkey, for the most part without effective occupational safety regulation.

One of the largest shipbreaking facilities in the world is in the coastal town of Alang, in Bhavnagar district, Gujarat, India. Alang is home to some 120 ship-recycling companies, employing more than 40 000 workers. 

Located in the nearby city Ahmedabad, Joydeep Majumder, a workplace noise and audiometry expert, working at the National Institute of Occupational Health, is familiar with conditions in Alang and identifies two key concerns. 

The first is the way work is organized, with workers exposed for prolonged periods to harmful noise. “Oxy-fuel torch cutters generally work in the same area all day and can be exposed to levels of sound exceeding 140 decibels for prolonged periods,” Majumder says. He has petitioned ship recycling companies to allow workers to change task or location on the work site, but with limited success.

The second concern is the lack of personal protection or rather the use of personal protection by workers. “Workers do not want to use ear plugs or ear muffs because they cannot hear each other and because of the humidity. They are sweating heavily and the ear plugs are uncomfortable. They also fear getting ear infections.”

“Relying on personal protective equipment should be a last resort.”

Manal Azzi

For Majumder improving awareness of the risks to which ship recyclers are exposed is key to improving the uptake and use of personal protection. “The majority of these workers are not even aware that they have a problem,” he says. “They typically come from rural areas and are surrounded by other hearing-impaired people and they are all shouting, and they do not realize what is happening until they go back to their families.” Majumder has been trying to increase awareness and encourage personal protection use by conducting regular training sessions on site with colleagues.

WHO’s Chadha agrees that raising awareness is vital. “The earlier workers become aware of the damage being done to their hearing, the more likely they are to protect themselves,” she says, emphasizing the importance of regular audiological assessment for early identification of onset of hearing issues. 

According to Rohit Agarwal, managing partner of Guideship Consulting Services, a company offering consultancy services to ship recycling companies in Alang, working conditions are improving, as companies strive to comply with the International Maritime Organization’s *International convention for the safe and environmentally sound recycling of ships*, referred to as the Hong Kong Convention, which includes provisions for hearing protection.

“Around 80 of the Alang yards have achieved Hong Kong Convention compliance certificates and more are undergoing audits for certification,” he says, arguing that the pressure for change is coming from responsible ship owners. “They not only sell their vessels to Hong Kong Convention-compliant yards, but appoint third-party supervisors to monitor activities and ensure standards are maintained.”  

Ship recyclers are also competing for European Union flagged vessels, which since 2018, can only be recycled in facilities that are compliant with the above-mentioned EU directive. To date, none of the Indian recyclers has been included.

For ILO’s Azzi, the conditions faced by India’s ship recyclers typify challenges faced by many of the estimated two billion people now employed in informal work, most of them in emerging and developing countries. “Improving workers’ hearing health and their health generally, depends on establishing safe and healthy working environments,” Azzi says. “Achieving that will require a transition to the formal economy.”

**Figure:**
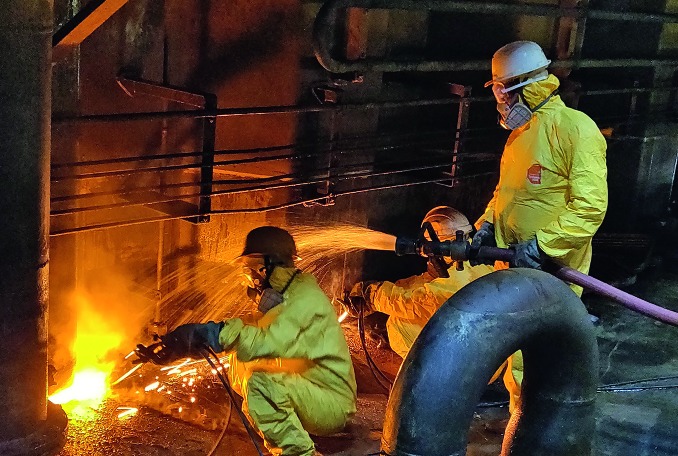
Recyclers cutting up a slop tank at the Alang Ship Recycling Yard, India.

Courtesy of Rohit Agarwal

**Figure:**
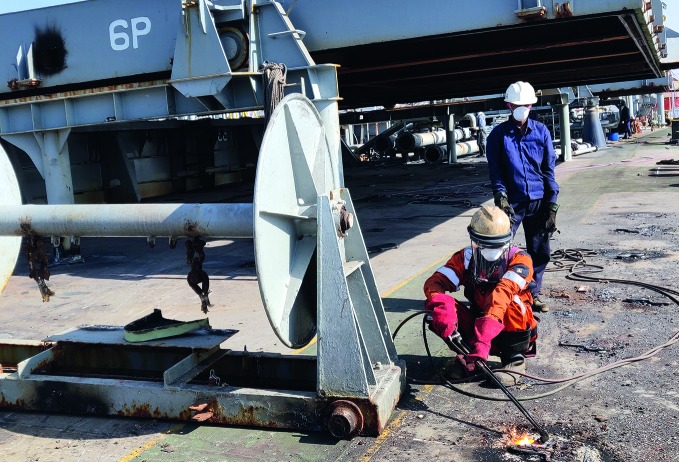
A torch cutter works on the deck of a ship at the Alang Ship Recycling Yard, India.

Courtesy of Rohit Agarwal

